# ‘Knocking on the Door, Saying, “Can We Come in?”’: Carers' Experiences of Engagement With Healthcare Workers in Inpatient Mental Health Services

**DOI:** 10.1111/jocn.70423

**Published:** 2026-07-03

**Authors:** Birhanie Mekuriaw, Natalie Ann Cutler, Tim Luckett, Jo River

**Affiliations:** ^1^ School of Nursing and Midwifery, Faculty of Health University of Technology Sydney Ultimo New South Wales Australia; ^2^ Department of Psychiatry, College of Health and Medical Science Dilla University Dilla Ethiopia; ^3^ Mental Health Drug and Alcohol Services Northern Sydney Local Health District Sydney New South Wales Australia; ^4^ Improving Palliative, Aged and Chronic Care through Clinical Research and Translation (IMPACCT), Faculty of Health University of Technology Sydney Ultimo New South Wales Australia

**Keywords:** caregivers, engagement, lived experience, mental health services, mental healthcare workers, recovery‐oriented practice

## Abstract

**Aim:**

This study explores carers' experiences of engagement with mental healthcare workers in public inpatient mental health services to better understand how carers can be optimally supported to contribute to recovery‐oriented care.

**Design:**

An exploratory qualitative descriptive study.

**Method:**

We conducted semi‐structured interviews with ten carers using a reflexive thematic analysis that emphasised systematic coding and reflexivity to enhance rigour.

**Results:**

Three overarching themes were identified, including: *Exclusion and tokenism*, which captures carers' experiences of limited inclusion and superficial participation in inpatient mental health services; *Fractured and medicalised system*, which highlights the disconnect between the rhetoric of recovery and the reality of highly medicalised and risk‐averse practice; and *Occasional care*, which describes genuine collaboration and shared understanding between carers and mental healthcare workers that are intermittently present, supporting trust and hope in some instances, but absent in others.

**Conclusion:**

Carers experience significant challenges when engaging with mental healthcare workers. The findings suggest the need for a genuine transformation of mental health systems and practice, with meaningful carer engagement central to relational recovery and recovery‐oriented care.

**Relevance to Mental Health Services:**

Findings from this study indicate that carers often lack consistent recognition and access to appropriate support, needed to fulfil their potential in supporting recovery. Strengthening formal support pathways for carers is therefore essential to promoting recovery that is relational, sustainable and shared.

**Patient or Public Contribution:**

A professional carer peer worker was consulted on the development of the study design and questions. Carers participated through in‐depth interviews.

## Highlights

- Advances understanding of relational recovery by demonstrating how carers' exclusion undermines recovery as a shared and relational process within inpatient mental health settings.
- Describes how fragmented, medicalised systems limit opportunities for sustained relational engagement between carers and mental healthcare workers, constraining relational recovery practices.
- Reinforces the need for global mental health services to embed meaningful, consistent carer‐mental healthcare worker relationships as central to recovery‐oriented practice.

## 1 Introduction

Carers play a critical role in supporting people with lived experience of mental health challenges (people with lived experience), providing emotional, practical and advocacy support that contributes to improving recovery outcomes (Mekuriaw et al. 2025). Carers may include family members, friends or other support persons who provide unpaid care to people with lived experience. Despite Australian national and state policies emphasising carers as partners in recovery‐oriented mental health care, many carers report exclusion from meaningful involvement within inpatient mental health services (Mercuri and Petrakis 2025). Such experiences disrupt the interconnected recovery journey of people with lived experience and carers themselves (Wyder et al. 2025).

Existing literature highlights that effective engagement between carers and mental healthcare workers is complex (Maybery et al. 2021). Despite a growing body of work emphasising the importance of trust, mutual understanding and recognition of carers' expertise in facilitating carer engagement (Mekuriaw et al. 2025), there remains a limited understanding of how carers' experience interactions with mental healthcare workers in inpatient mental health settings and to what extent their engagement supports carers in their caring role (Isaacs et al. 2025). This study addresses this gap by examining carers' experience of engaging with mental healthcare workers in public inpatient mental health services within the Australian healthcare system.

## 2 Background

Carer participation in recovery‐oriented care is a fundamental policy principle in contemporary mental health services, with the value of carers' contribution to recovery increasingly recognised (Mental Health Carers Voice 2021). Australian national and state‐level mental health frameworks have explicitly advocated the inclusion of carers as partners in mental health services to support a recovery‐oriented approach to care (Commonwealth of Australia 2013). Recovery‐oriented care draws on the contemporary ‘personal recovery’ paradigm used in mental health settings, which emphasises supporting people with lived experience to live connected, hopeful, meaningful and autonomous lives, with or without ongoing mental health symptoms, and recognises that recovery goals are subjectively defined by the person with lived experience, rather than clinically defined (Jordan et al. 2024). Recovery‐oriented care, therefore, is focused on the self‐defined recovery goals of people with lived experience (Harvey et al. 2023), operationalised through key domains, including promoting a culture of hope; engaging in person‐centred and holistic care; supporting personal recovery through partnership in care planning and the provision of strengths‐based psychosocial care (Harvey et al. 2023), and transparent communication and information for carers (Foster et al. 2016).

In Australia, nearly one million carers provide unpaid support to individuals with lived experience, which is an estimated contribution of time equivalent to $14.1 billion annually (Mental Health Carers Australia 2024) and represents more than 7% of the total annual economic cost associated with mental health conditions (Whiteford 2021). Given the major contribution of carers, engagement with carers in contemporary mental health settings is seen as a core and expected dimension of recovery‐oriented practice (Chatwiriyaphong et al. 2024). Australian frameworks for recovery‐oriented practice explicitly state the need for recognition and involvement of, and responsiveness to, families and carers, including provision of information, guidance and support (Commonwealth of Australia 2013). Carers not only have a role in supporting the recovery of people with lived experience, including in providing direct emotional and practical assistance, but they also support people with lived experience to navigate and engage with mental health services (Javed and Herrman 2017). In addition, carers serve as the primary advocates for people with lived experience, supporting self‐determined personal recovery and social and community inclusion (Whaley et al. 2024). Carer support has been associated with reduced likelihood of admission or readmission to hospital and improved quality of life for people with lived experience (Miles et al. 2020).

Wyder and Bland (2014) argue that carers experience distinct recovery journeys of their own, conceptualised as a form of ‘relational recovery’, in which carers' recovery is entwined with their caring role and with the recovery experiences of the people they support. In this context, relational recovery is seen as a shared and interconnected process between carers and individuals with lived experience (Wyder et al. 2022). Negative experiences with mental healthcare workers and mental health services, including a lack of effective engagement or inclusion, can undermine carers' capacity to support recovery and can also adversely affect the relational recovery journey of both carers and people with lived experience (Abou Seif et al. 2022). As such, there is increasing recognition that engagement with carers in recovery‐oriented practice, not only supports the recovery of people with lived experience, but is also vital to promote relational recovery of people with lived experience and their carers (Wyder et al. 2022). In this context, ‘engagement’ refers to the process of establishing and maintaining meaningful communication, involvement and collaboration, facilitated by relational, organisational and practical factors that support carers' involvement.

Despite policy support for carer involvement in recovery‐oriented mental healthcare, and clear recognition of the value of carers' contributions, carers still experience a lack of recognition for their role in supporting recovery (Maybery et al. 2021). Engagement of carers in recovery‐oriented practices is reported to be inconsistent, particularly in inpatient settings where services may have limited resources and prioritise efficiency and medicalised care over engagement with carers (Cartwright et al. 2024). Carers report a lack of meaningful inclusion in inpatient mental health services, including a lack of communication and information and involvement in core dimensions of care, including care and discharge planning (Israel et al. 2023). Exclusion of carers not only undermines recovery‐oriented care and recovery outcomes, but it can also reflect and reinforce professional hierarchies that privilege clinical recovery, which emphasises medical treatment and symptom reduction rather than relational and self‐determined recovery (Ziegenhagen et al. 2025) and can undermine the personal recovery journey of carers and people with lived experience (Wyder et al. 2022). In addition to exclusion, carers also report experiencing stigma from mental healthcare workers, which erodes carers' confidence, restricts their involvement and perpetuates social exclusion for both carers and the person they care for (Zwar and Hajek 2025). This stigma further operates as a barrier to carer engagement in recovery‐oriented care, constraining opportunities for partnership in care and weakening the relational recovery process (Zwar and Hajek 2025).

Despite a growing body of research on carers, little is known about how carers engage with mental healthcare workers in inpatient mental health settings and how engagement supports carers' roles and relational recovery. According to a relational recovery framework, effective engagement would involve collaborative, trust‐based and reciprocal processes (Wyder et al. 2025), which are operationalised through relational, informational and decisional dimensions of recovery‐oriented practice. These dimensions have been more clearly articulated in family‐centred care frameworks for mental health care, and include actions such as recognition of carers as integral and active partners in recovery; assessment of carer needs; provision of clear and transparent communication and information to carers; collaboration with carers in decision‐making, including care planning; and education and support for carers (Foster and Isobel 2018). However, to determine the quality of carers' engagement with inpatient mental health services, a more comprehensive understanding of carers' experiences is required. By exploring how engagement is enacted between carers and healthcare workers within recovery‐oriented public inpatient mental health care settings, the study is addressing an interactional gap in the literature. The context is relevant due to policy and practice expectations that public mental health inpatient services facilitate recovery‐oriented care through the meaningful involvement of carers. A more detailed understanding has the potential to inform measures within inpatient mental health services to improve carer engagement and related recovery outcomes.

## 3 The Study

Despite evidence supporting the benefits of carers' inclusion in recovery‐oriented mental healthcare, little is known about how carers engage with mental healthcare workers and services. It is also unclear which factors support carers' involvement in ways that enhance their capacity to promote the recovery of people with lived experience. A more comprehensive understanding of carers' experiences and needs, particularly in relation to engagement with mental healthcare workers, is required to inform measures within health services to improve carer engagement and related recovery outcomes, both personal and relational. This study sought to address this gap by exploring carers' experiences of engaging with mental healthcare workers in public, inpatient mental health settings, where healthcare workers seek to provide recovery‐oriented care. Specifically, the study examined factors that facilitate and hinder carer engagement with mental healthcare workers and mental health services.

## 4 Methods

### 4.1 Study Design

A qualitative descriptive study design, as outlined by Sandelowski (2010) and further explicated by Hunter et al. (2019), was employed to explore the experiences of caregivers engaging with mental healthcare workers. This methodology is a pragmatic approach that allows researchers to produce a detailed and accurate account that stays close to participants' words and experiences and is particularly useful when little is known about a topic (Villamin et al. 2025). Qualitative descriptive methods have also been aligned with constructionism, which recognises that participants' accounts are subjective, contextual and plural (Denzin et al. 2018). As such, a qualitative descriptive methodology provided a flexible approach that could adopt interpretive analytical methods while remaining close to descriptions of carers' subjective and contextual experiences in mental health services (Doyle et al. 2020). Qualitative descriptive design has been widely used in mental health research due to its suitability for producing clear, practice‐focused insights that can directly inform service improvements (Daliri et al. 2024).

At the initial stage of the study, a professional carer peer worker was consulted on the research focus and design to ensure it reflected carers' concerns regarding inpatient mental health services and that the findings would be relevant and meaningful to their needs. This helped us minimise imposing authors' assumptions based on their prior clinical experience. The study's methodology was evaluated against the Consolidated Criteria for Reporting Qualitative Research (COREQ) (Tong et al. 2007) (see SD‐3).

### 4.2 Recruitment

We employed both purposive and snowball sampling (Nyimbili and Nyimbili 2024). Information flyers were sent by email to peak carers' organisations within Australia with a request to promote the study to their members. Participants were eligible for the study if they were aged 18 or older, had experience providing unpaid care to a person with lived experience and had engaged with an inpatient mental health service in Australia within the past 10 years. This period was chosen as it followed the introduction of recovery‐oriented policies within Australian mental health services in 2013, a change that took several years to become fully embedded in practice (Commonwealth of Australia 2013). These criteria informed the recruitment and specifically targeted carers with recent experience of inpatient mental health services under a recovery‐oriented policy context. Efforts were also made to recruit participants with diverse age ranges, caring relationships and social backgrounds to capture a range of perspectives. Recruitment ceased following a research team discussion that the dataset provided sufficiently rich and meaningful accounts to address the research aims (Hennink and Kaiser 2022; Malterud et al. 2016).

### 4.3 Data Collection

Data were collected using semi‐structured, individual interviews conducted by the lead researcher, who has prior experience working with carers in mental health settings. This experience supported rapport building and sensitivity during interviews, but also required ongoing reflexive attention to how professional background, assumptions about carer experiences and familiarity with mental health practice may have influenced both data collection and interpretation. A systematic review, which informed interview questions of carer experience conducted by our team (Mekuriaw et al. 2025), as well as by consultation with a professional carer peer worker, who provided feedback on the study design and interview questions (See SD‐1). Following ethics approval, interviews were conducted with participant consent via an online platform (Zoom), and audio recorded to ensure data accuracy. In line with the ethics approved protocol, recordings were stored on a secure system and were accessible only to the research team. Data were collected between January and April 2025, with interviews ranging from 60 to 90 min.

### 4.4 Data Analysis

Interviews were automatically transcribed using Zoom's transcription feature. As required for ethical approval, transcripts were securely stored and de‐identified. Each transcript was then carefully checked and corrected against the original audio recordings to ensure accuracy and fidelity to participants' words. Data were analysed inductively using the reflexive thematic analysis approach designed by Braun and Clarke (2022).

Reflexive thematic analysis has been used previously with qualitative descriptive studies to explore and describe participants' experiences (Hunter et al. 2019). Although reflexive thematic analysis is commonly aligned with interpretive qualitative paradigms, its theoretical flexibility allows it to be applied at varying levels of interpretation. In this study, the thematic analysis was conducted descriptively (low inference), prioritising participants' explicit accounts while acknowledging the researcher's reflexive role in constructing meaning (Doyle et al. 2020). Reflexive thematic analysis has six steps (data familiarisation, generating codes, generating themes, reviewing themes, defining and naming themes and writing up the analysis) (Braun and Clarke 2022). The Relational Recovery framework (Wyder et al. 2022) was used as a sensitising concept during the analysis phase to support interpretation of the emergent themes, particularly in relation to the relational qualities of carers' engagement with mental healthcare workers, while ensuring that the analysis remained grounded in participants' own experiences (Wyder et al. 2022).

Coding was carried out iteratively, allowing codes to be developed, reviewed and refined throughout the analysis. Data were systematically coded using NVivo qualitative analysis software to support the organisation, management and analysis of the transcripts (Lumivero 2023). The lead researcher undertook primary coding, and reflexivity was supported through regular team discussions to critically examine how the researchers' perspectives may shape interpretation and to incorporate multiple viewpoints into the analysis. Researchers then collaboratively reviewed, refined and defined the emerging themes through an iterative process. Data analysis proceeded iteratively until the research team decided that sufficient depth had been achieved to support a coherent and meaningful interpretive account. Rigour was addressed through a reflexive thematic analysis approach (Braun and Clarke 2022), with iterative coding, ongoing immersion in the data and team discussions to ensure reflexivity and methodological transparency. Transparency was further maintained by documenting analytic decisions, and credibility was enhanced by grounding interpretations in participants' accounts through illustrative data extracts (see SD‐2).

### 4.5 Ethics Statement

The study was conducted in accordance with the Australian National Statement on Ethical Conduct in Human Research. Ethical approval was obtained from the University of Technology Sydney (UTS) Human Research Committee (approval number: ETH24‐9149). Utilising Zoom for conducting and recording interviews, as well as procedures for secure data storage and management, were reviewed and approved. All participants provided written consent after receiving information about the study and could withdraw at any time. To safeguard privacy, the data are anonymised to ensure the identities of participants, the person they care for and any service providers or services are protected. All data were securely stored on the UTS institutional data management platform (UTS Stash), in accordance with institutional and ethical requirements.

## 5 Findings

### 5.1 Participant Characteristics

Among a total of ten participants (*N* = 10), 80% identified as female (*n* = 8). Half of the group (50%; *n* = 5) were aged 50 years or over. The majority lived in the Australian state of Victoria (60%; *n* = 6), with the remainder living in the state of New South Wales. In terms of caregiving duration, 60% (*n* = 6) had been caring for at least eight years. In addition to engaging with inpatient mental health services (100%; *n* = 10), participants also reported engaging with community mental health services (70%; *n* = 7), rehabilitation services (40%; *n* = 4) and private mental health services (40%; *n* = 4). Participant characteristics are detailed in Table 1. Although participants had engaged in multiple service types, the study findings focus specifically on their experiences of engagement with public inpatient mental health services.

**TABLE 1:** Participant characteristics.

Variable	Categories	Number (*n*)	Percentage
Gender identity	Female	8	80
	Male	1	10
	Self‐described	1	10
Age group of participants (years)	20–29	2	20
	30–39	1	10
	40–49	2	20
	50–59	5	50
Relationship to care recipient	Parent	7	70
	Sibling	2	20
	Child	1	10
State of residence	New South Wales	4	40
	Victoria	6	60
Occupation	Professional (social worker, carer advisor, client service advisor, project manager)	4	40
	Student (college student)	4	40
	Volunteer (free service care providers)	2	20
Caregiving duration	2–4 years	2	20
	5–7 years	2	20
	8–10 years	6	60
Care recipients' age group (years)	16–20	2	20
	21–30	3	30
	31–40	2	20
	> 40	3	30
Mental health services accessed (more than one option available)	Acute inpatient	10	100
	Community	7	70
	Rehabilitation	4	40
	Private	4	40

### 5.2 Qualitative Findings

Three central themes were identified, capturing how participants experienced engagement with the mental healthcare workers in their role as carers. These themes were: *Exclusion and tokenism, Fractured and medicalised systems, and Occasional care*. When discussing engagement with mental healthcare workers, participants also discussed the context of this engagement with the mental health service environment and how this impacted their capacity to engage.

### 5.3 Exclusion and Tokenism

The theme *Exclusion and tokenism* captures participants' experiences of exclusion from, or tokenistic involvement within, inpatient mental health services. The theme captures carers' direct, relational experiences within interactions with mental healthcare workers and encompasses two sub‐themes: ‘Overlooked and excluded’, and ‘Token engagements’.

#### 5.3.1 Overlooked and Excluded

Many participants reported feeling overlooked and excluded, with little opportunity to connect with mental healthcare workers, particularly during extended waiting periods where no interim supports were provided, which appeared to produce uncertainty and contribute to disengagement. This was exemplified in one participant's experience of accessing inpatient mental health services:

So, for example, mental health services in the public system there's a long wait, and there's nothing really. There was no advice, guidance, support, or anything given in the interim, and there was a long time [*to the*] initial appointment. So, that time between the first contact and then the initial appointment was a long gap. (P9)

Even once carers engaged with mental health services, they still struggled to be heard, repeatedly ‘knocking on the door’ to participate, share their experience and seek support, yet their urgent needs were often overlooked. As one carer recounted:

It's us carers knocking on the door, saying, ‘Can we come in? Can we participate? Can we share our story? Can we get support?’. (P2)

This participant's account is arguably indicative of power disparities within mental health services, which left carers disempowered in their relationships with healthcare workers and pleading for inclusion, as opposed to being active partners in care.

This lack of inclusion was also indicated in participants' accounts of a lack of information or education provided by mental healthcare workers, which significantly increased stress and made their caregiving role more difficult. Carers described being structurally invisible in mental health services, being routinely and systematically overlooked and excluded from key mental healthcare processes, despite identifying themselves as central to care planning and recovery. For example, participants reported exclusion from care planning, even when their ongoing involvement was critical to their implementation and success. One carer described being left out of these processes despite playing a pivotal role in making them work:

Carers are often excluded from, you know, the well‐being plan, the safety plan, the recovery plan, even though we're pivotal to all of that, working out and being successful. (P2)

Instead, as one participant put it, ‘rather than involvement,’ tasks are ‘just delegated to me’.

While information and participation were perceived as vital to the carer role, the primary need expressed by many carers was for ongoing, practical and emotional support as a central requirement for role enactment:

I was hoping that the service could give me some support and help me through this [*carer role*] by providing some education, providing some support, and I could support my family member…but unfortunately, I didn't have any support from them, and I just got more concerns, and I became more stressed. (P8)

The neglect of carers' support needs was perceived as harmful to carers' wellbeing and could affect the care they provided, highlighting the need for carer‐focused support within care systems. Carers also perceived a lack of interest from mental healthcare workers in carer wellbeing and recovery, implying marginalisation and lack of recognition:

It's very rare that I've been asked as a carer, How are you? Are you OK? […] Let alone my own recovery journey, and my own trauma because of my caring experiences, that's completely ignored. (P3)

Participants indicated that experiences of being overlooked and excluded were compounded for carers with intersectional identities, for example, LGBTIQA+ carers or carers from culturally diverse backgrounds, as mental healthcare workers failed to understand or attend to the specific challenges they faced. For example, one participant noted:

These workers couldn't fully understand the issues they [*LGBTIQA+ carers*] were experiencing. That's why I believe it's important for mental health institutions to employ culturally diverse staff and provide cultural competency training. (P10)

Carers felt overlooked due to limited attention to their wellbeing and a lack of LGBTIQA+‐specific cultural competency, resulting in their identities and needs being insufficiently recognised within inpatient mental healthcare settings.

Carers' accounts revealed a systemic pattern of marginalisation within inpatient mental health services, where limited engagement and exclusion from decision‐making conveyed that their knowledge, wellbeing and identities were not valued. Rather than being recognised as partners in care, carers were positioned as invisible, experiencing a lack of support that impacted their wellbeing and negative experiences could be further compounded for those with intersectional identities.

#### 5.3.2 Token Engagements

Data indicate that in some instances, engagement did occur. However, it was perceived by carers as tokenistic and inadequate to meet their needs. One participant described the gap between the rhetoric of inclusion and the absence of meaningful involvement:

And it's very tokenistic. It's like, … ‘I'll call you, and you have a 10‐minute chat.’ (P2)

Participants also noted that mental healthcare workers often failed to meaningfully involve carers in key decisions, prioritising procedural control over relational engagement. One participant noted:

They organise the whole, or they run the whole procedure in a way, that they don't care about how people feel. They [*mental healthcare workers*] are very controlling, and they don't respect the family, the carers, or the patients' concerns or decisions. (P4)

Similarly, some participants described minimal levels of engagement, including passive receipt of formal care‐planning notifications provided without sufficient explanation, resulting in a lack of understanding of treatment processes and minimal involvement in care:

They sent me a notice or a form for care planning. I've only ever received a notice about the treatment order, so what do they plan to do with the next one? How many injections is he going to have, for how long? So I don't know really what the psychologist is doing with him, and the sort of therapy she gives him. (P1)

Information provision was largely generic and standardised, giving the impression of engagement while failing to support meaningful involvement. The lack of individualised, family‐focused communication meant that carers were informed about care rather than engaged in it, limiting their ability to participate substantively in decision‐making:

They [*mental healthcare workers*] are very good at giving you lots of information. But it's global, it's universal, it's generic information, right? For my son and our particular family situation, it was not incentivised information. It was ‘here is a blurb about psychosis, or about medication side effects, or a safety plan’, which tends to be very generic as well, so it's not individualised and it's not family‐focused either. (P2)

Other carer participants encountered conflicting information from different mental healthcare workers, indicating poor communication and information practices:

I think there was an inconsistency between speaking to one professional versus talking to another, and one would tell me one thing or tell me that I was eligible for these sorts of supports, and then the next would tell me that I wasn't. I think that was probably the main thing that made the communication a struggle because nobody seemed to be on the same page. Everybody was saying different things. (P7)

As this quote illustrates, inconsistent communication and information made it difficult for carers to know what support they were entitled to.

One participant reflected on the uncertainty she experienced due to a lack of clear and specific communication and information about her son that was tailored to her caring needs:

If someone said, ‘he is going to need full‐time care’, at least I'd have some clarity. […] If someone told me what was really going on in his head, I would understand. If they told me face‐to‐face, we could discuss it, talk it through, and grieve together. (P1)

This participant's account also flagged the potential impact of a lack of consistent information on carer recovery, such that it prevented her from grieving the loss of a life she had expected for her son.

Carers' interactions with mental healthcare workers often felt superficial, providing involvement without meaningful information or capacity to influence. Generic, inconsistent and impersonal communication left carers disempowered. Engagement was thus experienced less as collaboration and more as exclusion masked by formal procedures.

### 5.4 Fractured and Medicalised Systems

This theme reflects the broader structural and organisational context in which interactions occur, and describes participants' experiences of mental healthcare worker engagement in the context of a fractured and medicalised healthcare system, captured in the three subthemes of ‘Fragmented and poor‐quality care’, ‘Deficit‐focused’ and ‘Medicalised approach’.

#### 5.4.1 Fragmented and Poor‐Quality Care

Participants described how their engagement with mental healthcare workers was marked by significant gaps in both the quality and continuity of services, with inconsistent care experiences, poor coordination between services and poor‐quality care, including the environments in which care was provided.

Some participants noted that poor communication between mental healthcare workers themselves and inadequate record‐keeping undermined continuity of care. As a result, carers often had to retell their story to each new mental healthcare worker, which was perceived as being disruptive to building a trusting therapeutic relationship. As one carer explained:

The histories are not often recorded appropriately, so when you go to a new [*mental healthcare worker*], even if your file or your case is transferred, you still have to retell all of your stories. (P5)

Another participant noted that referrals were often occasional and inconsistent, emphasising that support ‘needs to be embedded within the mental health system.’ This highlights how reliance on external referrals can fragment carer support and limit continuity across the care journey:

Occasionally, had a referral to a social worker, who then referred me to a mental health carers' carer gateway and other external organisations, but those support systems need to be embedded within the mental health system, and we need to be followed through the entire journey, not just when my son turns up to the emergency department or has an admission. (P7)

Most participants also noted a lack of coordinated follow‐up and ongoing support from mental healthcare workers after discharge, which inadvertently shifted responsibility for follow up care onto carers. This was experienced as an abrupt withdrawal of care that disrupted continuity and left carers feeling abandoned, struggling to manage the day‐to‐day care needs of the person they cared for. As one participant described:

So, yeah, so [*the mental healthcare workers*], they completely stopped the contact and like I said—I've forgotten now—like since my brother had that community treatment order ever since he was discharged, ‘they just wiped their hands of him’. (P3)

Several participants described their experience of engaging with inpatient mental health services as overwhelmingly negative and difficult to navigate, positioning the system as more challenging than other care sectors, where care pathways were unclear and support was inconsistent.

Overall, in one word, probably a negative experience. I say ‘negative experience’ because I've been a carer in aged care as well, and I thought that was challenging, but the mental health system, I think, it's much harder to navigate. (P3)

Participants viewed negative experiences with mental healthcare workers, not as minor interpersonal issues, but as a fundamental failure of care that undermines service quality. One participant questioned the suitability of some mental healthcare workers for their role, given the relational demands of working with carers and people with lived experience, where empathy was viewed as essential for therapeutic engagement and trust:

I have to wonder why the people who are working in the industry why they're even there, you know, if they can't offer even a fraction of compassion to people who need it, then they could be working anywhere. They could be working at a bank or somewhere that doesn't require any sort of empathy or compassion. (P3)

Another participant pointed to the poor quality of the mental healthcare environment, describing the service as unclean and poorly maintained:

It was also not my expectation, I guess there was like…where he [*person with lived experience*] was staying was not nice. Not a nice place, it was actually the wall, the paint coming off the walls…. The floors were filthy, and the bathroom smelled. I had to change the sheets, and it's a public hospital. So, I felt those surroundings were not that nice. (P1)

Poor environments were sometimes construed by participants as implying a lack of care and respect for people with lived experience, thereby undermining trust, safety and the therapeutic relationship. Indeed, one participant noted the poor quality of the physical environment, suggesting that substandard conditions could exacerbate distress for people with lived experience and their carers.

Participants' accounts indicate that mental health public inpatient services are experienced as fragmented and impersonal, where poor coordination, limited empathy and abrupt withdrawal of support erode continuity of care and left carers feeling abandoned and burdened with navigating care alone.

#### 5.4.2 Deficit‐Focused

Despite the hopeful principle of promoting ‘personal recovery’ espoused by mental health services, participants reported that this recovery rhetoric often failed to translate into meaningful recovery‐oriented care. Instead, mental healthcare workers were frequently deficit‐focused, rather than recovery‐oriented, prioritising limitations over supporting the persons' strengths and recovery goals:

I think one of the challenges with some clinicians is that they tend to focus too much on the problem itself. The conversation often revolves around what is wrong or what the issue is at the moment. But I feel like there is not always enough of a strength‐based approach, really looking at what the client is already doing well, identifying their existing strengths, and building on those. That way, the focus shifts toward empowering the client, rather than just highlighting the problem they're facing. (P10)

Some participants described mental healthcare workers as prioritising risk management and containment over supporting individual recovery goals, illustrating a deficit‐focused approach to care.

It was all about control, about trying to fix the person rather than working with my son to say, ‘What are your goals and needs?’ It was problem‐focused rather than expansive and creative, and controlled by my son. It was done to him. It was more about making sure he wasn't readmitted or didn't hurt himself or other people, really risk‐averse rather than [*recovery*] focused. (P2)

As these data show, mental health care was frequently deficit‐focused, with mental healthcare workers prioritising risk management and control over recognising strengths and supporting personal recovery goals. This approach left a person with lived experience feeling disempowered and excluded from genuinely collaborative, recovery‐focused care.

#### 5.4.3 Medicalised Approach

In addition to being deficit‐focused, participants frequently characterised inpatient mental health services as overly focused on clinical approaches, particularly psychiatric diagnosis and medication and restrictive practices. This was often at the expense of broader recovery‐oriented dimensions, including the meaningful inclusion of carers in care planning and decision‐making. A medicalised focus also limited carers' opportunities to engage as active partners, reinforcing feelings of marginalisation and exclusion during the inpatient journey.

Some participants described this medicalised approach as reducing the person with lived experience to a set of symptoms to be managed, rather than recognising and understanding them as a whole person. As explained by one participant:

Medication is not always the answer; it is not really suited to everybody. You know you've got someone coming in with an anxiety issue and having panic attacks, and you're recommending mindfulness and meditation, which is important, but why medication? You know, a lot of the psychiatrists just think that giving medication fixes everything. That's not reality. (P9)

As this participant's account indicates, a focus on medical treatment could lead to a lack of attention to the recovery needs of people with lived experience, which in turn undermines support for carers' recovery.

Some participants reported fear and uncertainty when observing medication side effects, particularly when they perceived a lack of responsiveness to side effects by mental healthcare workers. One participant described concern after the person they cared for became increasingly fatigued following medication initiation, questioning whether the treatment was beneficial or harmful:

I had a lot of concerns when they prescribed the medication. I was very worried when I saw the side effects of medication…because she is a roommate, and she became very lazy and more tired… So my concern was if the medication is making her better or making her worse? And I talked to the psychiatrist and the psychologist. They just said, ‘OK, keep on going’. (P4)

Negative experiences with medication could lead to carers to position themselves as critical, rather than collaborative, participants in care. While some participants lamented the predominantly medicalised focus of inpatient mental health services, others supported the use of medication to promote the safety of the person with lived experience and those around them:

Well, I think the biggest asset, the biggest thing, is that they are keeping him on medication, which keeps his moods stable. At some level, so he is not going to hurt himself or me. That's important. That's probably all they are doing, and that is all the psychiatrist does. (P1)

As this quote suggests, even those carers who supported medication use expressed dissent about a purely medicalised focus to care.

Some participants also described coercive practices such as seclusion, restraint and forced medication, which they experienced as traumatic. These carers perceived coercive practices as an extension of a medicalised approach that was focused on control and risk management rather than dignity, autonomy and rights‐based recovery. As one participant stated:

I saw how they treated him every single day…Shackled to the bed for hours, locked in a tiny white room overnight, repeatedly restrained and injected to keep him quiet, always watched by security, His freedom was completely curtailed, and as a carer, witnessing that level of coercion was incredibly distressing. (P2)

As this account shows, this carer perceived restraint as a means of silencing the person with lived experience, limiting meaningful participation in care.

Taken together, these sub‐themes suggest that fragmented and medicalised service systems constrained relational recovery by limiting trust, reciprocity and collaboration in carers' engagement. Deficit‐focused and at times coercive practices further disrupted relational processes, with some carers describing experiences as distressing and dehumanising.

### 5.5 Occasional Care

This final theme described occasional positive experiences of care that contrasted with the largely negative experiences described in the previous two themes.

Some participants reported active inclusion in care planning through collaborative relationships with carers and mental healthcare workers. Shared discussions and goal‐setting enabled carers to participate meaningfully in mental health care. One participant explained how a mental healthcare worker facilitated joint reflection, supporting partnership‐based care planning:

I wasn't making decisions directly, like about medication, but my mum, our counsellor [*mental healthcare worker*], and I would work together. The counsellor would offer suggestions, and we'd talk through them as a team, setting goals and deciding how we wanted to move forward together. (P6)

Another participant referred to their consistent inclusion in regular care meetings that enhanced their engagement with mental healthcare workers as part of the team providing inpatient care:

As a carer, there would be meetings fortnightly, which would be with my sister's care team, so her mental healthcare team, and that's where I would be informed of her progress and any changes being made to her care plan, things like that, and I found that very helpful. (P7)

One participant reflected on their experience of sustained relationships with mental healthcare workers, who attended to their diverse support needs beyond clinical support, encompassing assistance with employment, education and navigating financial systems:

We've got a long‐term practitioner [*mental healthcare worker*] who's supported us through victim of crime processes, but also supports us through work and study as well. And we've had long‐term support that we've had for over 7 years, and which is really helpful, and a Medicare rebate is available as well. (P5)

While these positive experiences indicate that involvement of carers in inpatient mental health services is possible within the current remit of mental healthcare workers, data indicate that they were not the norm, with positive experiences less frequently reported. Notably, this suggests that little to no structured action has been taken to support carers in line with relational recovery.

## 6 Discussion

This qualitative descriptive study sought to examine carers' experiences of engaging with healthcare workers within public inpatient mental health services. Three overarching themes capture largely negative experiences of carers' engagement with mental healthcare workers, characterised by exclusion, tokenistic engagement and fragmented and medicalised service delivery, although occasional positive experiences of engagement were reported. These findings are important as they emphasise a persistent disconnect between policy directives and the lived reality of mental health service delivery, particularly in terms of realising effective engagement that attends to relational recovery.

Despite more than a decade of policy commitments to carers' inclusion in mental health care (National Mental Health Commission 2024), our findings indicate that carers continue to experience exclusion and tokenism in inpatient mental health services. These findings are consistent with other studies (Abou Seif et al. 2022), which found that when engagement did occur, it was often experienced by carers as tokenistic, with minimal, generic and conflicting information provided and a lack of genuine involvement in decision‐making. Data from the current study indicate that experiences of exclusion and tokenism leave carers feeling locked out of services, unable to receive information, support or care. These experiences can be understood as a manifestation of marginalisation within asymmetrical service power relations, where mental healthcare workers' privilege arguably overrides lived expertise (Brown 2026). Findings indicate that these experiences compromised carers' relational recovery and rendered some carers vulnerable to emotional stress in addition to their usual caregiving burden.

The current study also highlighted how carers experienced inpatient mental health services as fragmented and of poor quality. Indeed, findings show that carers perceived services as difficult to navigate and sub‐therapeutic environments, which undermined trust in mental healthcare workers and services and impacted personal and relational recovery. These experiences are congruent with broader critiques of mental health services as fragmented, crisis‐driven and poor quality (Isaacs et al. 2024). However, our findings indicate that fragmentation and poor quality services were not only experienced as service failures but also shaped carers' perception of mental health systems as a whole, leaving them feeling excluded, unsupported and disoriented with nowhere to turn.

Participants in our study reported that services were highly medicalised, deficit‐focused and risk‐averse rather than recovery‐oriented, which could increase reliance on coercive practices. While critiques of medical dominance in mental health care are well established (Rowland 2024), this study extends understanding of how these systemic orientations were experienced relationally and emotionally by the carers and the person they care for. As this study shows, coercive practices were not only perceived as traumatic to people with lived experience but also to carers who could find it highly distressing to witness. A lack of consideration for medication safety was also raised by carer participants, which is supported by quantitative research indicating that a significant proportion of care recipients experience a lack of medication monitoring (Ayre et al. 2023). However, this study adds qualitative depth by illustrating how carers experience medicalisation and coercive practices as a source of harm, particularly in the contexts where their concerns were perceived as minimalised by mental healthcare workers. Given that relational recovery is supported by trust in services (Wyder et al. 2022), experiences of medicalised and coercive practices were perceived as undermining trust and, at times, actively harmful to personal and relational recovery (Reid et al. 2025).

Beyond medication, participants highlighted a broader absence of psychosocial and relational supports. This absence signals a structural misalignment between the ideals of recovery‐oriented frameworks for practice (Commonwealth of Australia 2013) and healthcare workers' real‐world practice in inpatient mental health settings. Importantly, findings indicate that practice currently remains organised around a biomedical approach, which privileges medical treatment for symptom reduction and risk management, rather than being oriented to the recovery needs of carers and people with lived experience (Inta et al. 2026). The challenge is not unique to Australia, as similar critiques have been levelled in the United Kingdom, Canada and the United States (Patel et al. 2023). However, as this study shows, this arguably creates a dissonance for carers, who are aware of policy directives and practice frameworks to promote personal recovery, but continue to experience frontline mental health service delivery shaped by biomedical reductionism, thereby leading to a sense of disillusionment with mental healthcare workers and services and their capacity to provide compassionate and recovery‐oriented care.

Although occasional positive experiences of care were reported by participants, including clear communication, involvement in care planning and continuity of care, these often arose within an overall context of negative experiences. Indeed, findings indicate that carers' engagement with mental healthcare workers could be experienced as lacking compassion or concern for carers' support needs. The occasional positive encounters reported by carers in this study, however, reveal the potential for more inclusive and collaborative engagement within inpatient mental health services and further research might explore the drivers of positive and negative care interactions.

As this study shows, the marginalisation of carers in healthcare relationships not only disrupts the reciprocal and relational dimensions essential to carer recovery (Wyder et al. 2022), but can also exacerbate distress and create what has been described as ‘surplus suffering’ (James and Clarke 2001). While the concept of surplus suffering has usually been used to describe additional suffering experienced by people with a health condition due to negative health service experiences (Burke et al. 2018), our study shows that carers can also experience surplus suffering related to a lack of inclusion and support, which creates an additional burden of stress and distress beyond their usual caring role. Surplus suffering refers to the additional burden arising from systemic practices that invalidate carers' contributions and limit their participation, creating a cumulative layer of distress beyond the inherent challenges of the caring role. Indeed, rather than being supported in their own relational recovery journey, negative experiences of engagement left carers occupying a liminal space within the mental health system, suspended between recognition of recovery at the policy level and invisibility in practice.

Findings also indicated that experiences of being excluded were compounded for those carers with intersectional identities, including LGBTIQA+ carers and those from culturally and linguistically diverse backgrounds. Carers experienced additional barriers to engagement with mental healthcare workers stemming from cultural insensitivity and a lack of representation in inpatient mental health services. These findings resonate with intersectionality analyses of mental health care, which illuminate how multiple marginalised identities amplify and compound systemic exclusion of carers and the person they care for in mental health care (Faissner et al. 2024). Intersectional analyses of diverse mental health carer groups remain limited in Australian research. Future inquiries should prioritise understanding how structural and intersecting inequalities shape carer and mental healthcare worker engagement.

These findings reflect persistent challenges in achieving truly inclusive, empathetic and partnership‐based carer engagement in inpatient mental health care, where carers' expertise and concerns are meaningfully recognised (Mercuri and Petrakis 2025). The lack of care coordination and follow‐up after discharge is particularly concerning in this study, which contradicts the recovery‐oriented principles of continuity and predictability (Khatri et al. 2023). In response to this gap, as this study shows, carers often assume unpaid roles as advocates and compensate for structural deficiencies. However, it is important to acknowledge that our study focuses on public sector inpatient settings, which might differ considerably from community‐based or private healthcare contexts in terms of structure and opportunities for carer involvement. Adopting a longitudinal and mixed‐method approach in future research across a range of contexts would support understanding of how carer engagement evolves across the care transition from inpatient care to community‐based care.

## 7 Conclusion

This study illustrates how carers' experiences of engaging with mental healthcare workers, and by default, the mental health system, are shaped by a complex interplay of mental healthcare worker practice and mental health systemic factors, which often leave carers without adequate involvement, support or care that is genuinely (rather than rhetorically) recovery‐oriented. Carers' experiences were predominantly characterised by exclusion, tokenism, fragmented, poor quality and medicalised care that undermined personal and relational recovery. The findings reveal a mental health system that places considerable strain on carers. Carers' experience of being excluded, overwhelmed and distressed by deficit‐focused and medically oriented mental health services creates a ‘surplus suffering’ beyond their caring role. However, occasional positive instances suggest that more inclusive, integrated, recovery‐oriented care is possible. These findings underscore the urgent need for recovery‐oriented practices that are inclusive, relational and responsive to carers' needs, recognising their central role in the recovery journey and safeguarding carers' wellbeing as a critical component of sustainable mental health care and recovery.

## 8 Practice Implication

Embedding carers as active partners in care is required to address the relational recovery‐oriented practice gap. An authentic and genuinely recovery‐oriented mental health service, one that moves beyond rhetorical commitments and actively supports carers and people with lived experience, would be pivotal in bridging the current gap between policy directives and healthcare practice. Mental healthcare workers, including nurses, can take steps immediately to improve carer experience, including by recognising carer expertise and meaningfully involving carers in recovery‐oriented practices; engaging in consistent, clear and empathic communication with carers; and adopting a strengths‐ and rights‐based approach to care.

However, reform of services cannot sit with mental healthcare workers alone, and structural change is required to shift from medically oriented services to recovery‐oriented care, where non‐biomedical supports and diverse personal recovery pathways are actively supported. This would include structured models of carer involvement embedded in routine practice, with clear communication and information‐sharing protocols and formalised processes for carers' participation in care planning, discharge planning and post‐discharge follow up to ensure consistent engagement across the care continuum.

Enhancing workforce diversity is also essential to meet the needs of different carers and provide tailored mental health services. A trauma‐informed approach to mental health care, recommended by both national and international frameworks, could provide a more compassionate and inclusive foundation for engaging carers and eliminating coercive practices (Melillo et al. 2025). Integrating carers' experience into service quality assessment, as vital performance indicators, might also strengthen monitoring mechanisms and ensure support for carers. These reforms align with global mental health priorities outlined by the World Health Organization (2021), which calls for a shift from a reactive model to a preventive and community‐based mental health system.

## Author Contributions

All authors contributed to the conception and design of the study. B.M. conducted the participants' recruitment and carried out the interviews, with periodic consultation from J.R. and N.A.C. All authors participated in coding the transcript. B.M. conducted the initial coding of the data, and J.R. evaluated the coded data to ensure rigour. B.M. was responsible for conducting a qualitative analysis and generating the preliminary themes in consultation with all authors. B.M. drafted the initial version of the manuscript, and all authors contributed to critical revisions and approved the final version.

## Funding

The authors have nothing to report.

## Disclosure

The authors confirm no existing or potential conflicts of interest or financial benefits associated with this article.

## Conflicts of Interest

The authors declare no conflicts of interest.

## Supporting information


**Supporting Document S1.** Interview guide used during data collection.

**Supporting Document S2.** Evidence of rigour.

**Supporting Document S3.** COREQ checklist.

## Data Availability

Data supporting the findings of this study are available from the corresponding author.
