# Supplementary material for: ‘Knocking on the Door, Saying, “Can We Come in?”’: Carers' Experiences of Engagement With Healthcare Workers in Inpatient Mental Health Services
Source: J Clin Nurs. 2026 Jul 3;35(11):5044–57. doi: 10.1111/jocn.70423 (PMC13629246; doi:10.1111/jocn.70423)
Supplement: Supplementary file 2 [file JOCN-35-5044-s002.docx]

**Supporting Document 1 (SD-1): Interview Guide Used During Data Collection**

**Topic areas and questions**

Topic area questions are based on a systematic review of the literature and on key areas of concern or gaps in information regarding carers' experiences.

**Engagement with healthcare workers**

- What are your experiences of engaging with mental healthcare workers in mental health settings?
- How have mental healthcare workers involved you in supporting the person you care for in mental health settings?
- How has your experience of engaging with healthcare workers impacted your wellbeing and your role as a carer?
- In your view, how well do the mental healthcare workers keep you informed about the person you care for?
- Is there an aspect of care, e.g., care planning, discharge planning, where you need more support and guidance, if any?
- Do you find that mental healthcare workers are accessible when you want to reach them for questions, advice, or emergencies?
- Are there any specific barriers that make it difficult for you to access and communicate with mental healthcare workers?

**Recovery-oriented practice**

- Are you familiar with the concept of personal recovery, and if so, what does this mean to you?
- Have healthcare workers discussed the idea of personal recovery with you, and if so, how did they describe it?
- In what ways has engaging with healthcare workers supported you to be recovery-oriented in your carer role, if at all?
- What challenges have you faced in supporting recovery, and how have healthcare workers supported/not supported you with this challenge?

**Final questions**

- What changes would you like to see, if any, in the way mental healthcare workers engage with carers?
