# Supplementary material for: ‘Knocking on the Door, Saying, “Can We Come in?”’: Carers' Experiences of Engagement With Healthcare Workers in Inpatient Mental Health Services
Source: J Clin Nurs. 2026 Jul 3;35(11):5044–57. doi: 10.1111/jocn.70423 (PMC13629246; doi:10.1111/jocn.70423)
Supplement: Supplementary file 3 [file JOCN-35-5044-s003.docx]

**Supporting Document 2 (SD-2): Evidence of Rigour**

| **Domains** | **Explanation** |
| --- | --- |
| Credibility | - We reviewed the transcripts together and addressed inconsistencies as a research team. |
| Dependability | - We produce verbatim transcripts of all interviews. Preliminary codes and themes were generated and refined through several iterations until we established the final codes and themes - Codes and themes were repeatedly reviewed, refined, and revised through close and sustained engagement with the raw data, allowing patterns and meanings to be progressively clarified. |
| Transferability | - Each theme is well highlighted by including participants' quotes and comprehensive details. |
